# Diffuse goitre enlargement after immunotherapy for non‐small cell lung cancer

**DOI:** 10.1002/rcr2.866

**Published:** 2021-10-12

**Authors:** Kohei Fujita, Norio Araki, Tadashi Mio

**Affiliations:** ^1^ Division of Respiratory Medicine, Center for Respiratory Diseases National Hospital Organization Kyoto Medical Center Kyoto Japan; ^2^ Department of Radiation Oncology National Hospital Organization Kyoto Medical Center Kyoto Japan

**Keywords:** goitre enlargement, immunotherapy, nivolumab, PD‐1, thyroid

## Abstract

It is well known that a phenomenon called hyperprogressive disease (HPD) often occurs during immunotherapy with immune checkpoint inhibitors. In the present case, we experienced a case of HPD in a potential metastatic thyroid tumour during immunotherapy. HPD can be life‐threatening depending on where it appears, so clinicians need to be careful.

## Graphical abstract

We experienced a case of hyperprogressive disease (HPD) in a potential metastatic thyroid tumour during immunotherapy. HPD can be life‐threatening depending on where it appears, so clinicians need to be careful.

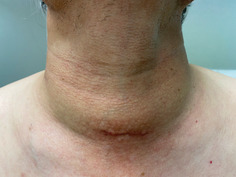

## CLINICAL IMAGE

**FIGURE 1:**
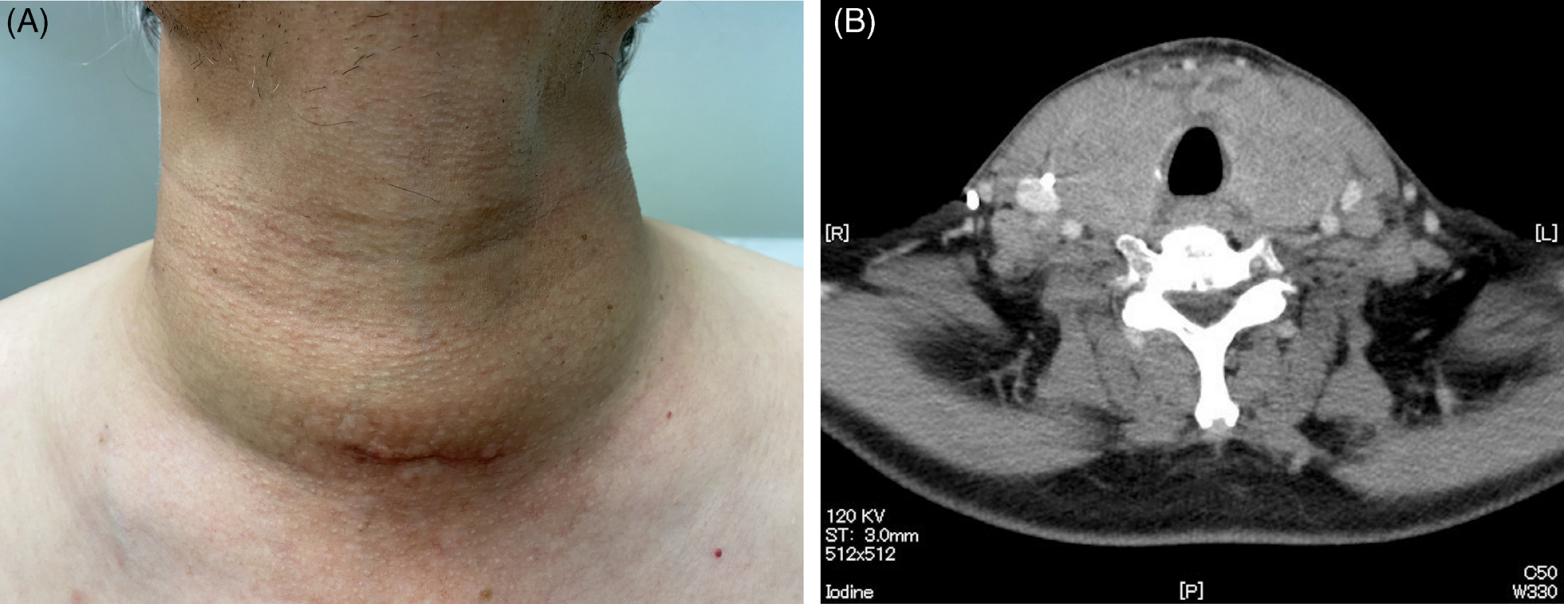
(A) Photography of the neck shows diffuse goitre enlargement. (B) Contrast‐enhanced computed tomography of the neck and chest revealed diffuse goitre enlargement

**FIGURE 2:**
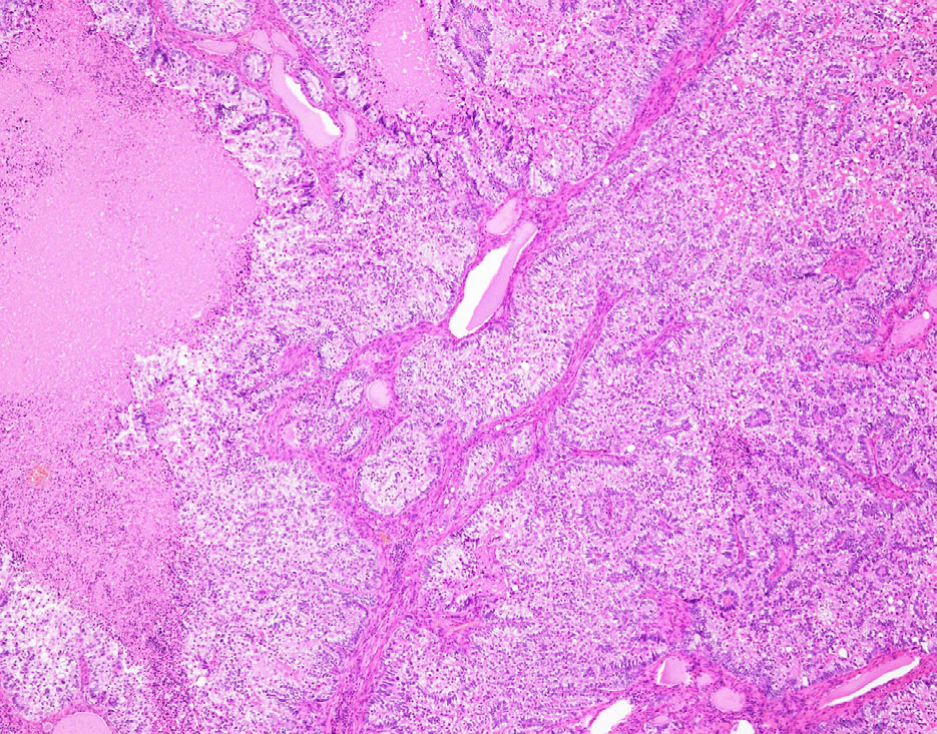
A biopsy of the thyroid tissue proved to be a metastasis of lung adenocarcinoma

A 58‐year‐old man with advanced lung adenocarcinoma presented to our hospital with a diffuse goitre enlargement with rapid increase in size within 7 days (Figure 1A). He had been diagnosed with stage 4 lung adenocarcinoma with bone and brain metastases. His adenocarcinoma had no driver oncogene alteration and showed less than 1% of PD‐L1 expression. He had received a cycle of immunotherapy with combination of nivolumab and ipilimumab for his lung adenocarcinoma just 2 weeks ago. At the time of consultation, there was no fever or dyspnoea, and no spontaneous pain or tenderness in the enlarged goitre. Thyroid function was within normal range. Contrast‐enhanced computed tomography of the neck and chest revealed diffuse goitre enlargement (Figure 1B). A biopsy of the thyroid tissue was performed and proved to be a metastasis of lung adenocarcinoma (Figure 2). In addition to changing the treatment regimen for lung cancer, he received palliative radiation therapy to prevent airway narrowing due to thyroid gland enlargement. This phenomenon is suspected to be hyperprogressive disease (HPD) caused by immunotherapy. Recently, increasing evidence shows that immunotherapy with anti‐PD‐(L)1 antibodies is associated with HPD.^1^ Previous study shows that heavy smoking, very low PD‐L1 expression and multiple metastases are risk factors for the development of HPD.^2^ The present case exhibits these risk factors. Clinicians must keep in mind that, during immunotherapy, HPD can occur even in potential new metastatic sites.

## CONFLICT OF INTEREST

None declared.

## AUTHOR CONTRIBUTION

Kohei Fujita treated the patient as the attending physician and drafted and revised the manuscript. Norio Araki performed diagnostic imaging and radiotherapy. Tadashi Mio performed the treatment. Norio Araki and Tadashi Mio revised the manuscript. All authors approved the manuscript.

## ETHICS STATEMENT

The authors declare that appropriate written informed consent was obtained for the publication of this manuscript and accompanying images.
